# Real Life Clinical Management and Survival in Advanced Cutaneous Melanoma: The Italian Clinical National Melanoma Registry Experience

**DOI:** 10.3389/fonc.2021.672797

**Published:** 2021-07-08

**Authors:** Anna Crispo, Maria Teresa Corradin, Erika Giulioni, Antonella Vecchiato, Paolo Del Fiore, Paola Queirolo, Francesco Spagnolo, Vito Vanella, Corrado Caracò, Giulio Tosti, Elisabetta Pennacchioli, Giuseppe Giudice, Eleonora Nacchiero, Pietro Quaglino, Simone Ribero, Monica Giordano, Desire Marussi, Stefania Barruscotti, Michele Guida, Vincenzo De Giorgi, Marcella Occelli, Federica Grosso, Giuseppe Cairo, Alessandro Gatti, Daniela Massa, Laura Atzori, Nicola Calvani, Tommaso Fabrizio, Giuseppe Mastrangelo, Federica Toffolutti, Egidio Celentano, Mario Budroni, Sara Gandini, Carlo Riccardo Rossi, Alessandro Testori, Giuseppe Palmieri, Paolo A. Ascierto, the Clinical National Melanoma Registry Study Group at the Italian Melanoma Intergroup, Maddalena Cespa

**Affiliations:** ^1^ Istituto Nazionale Tumori IRCCS Fondazione G. Pascale, Napoli, Italy; ^2^ Dermatology Department, Azienda Sanitaria Friuli Occidentale, Pordenone, Italy; ^3^ Istituto Oncologico Veneto IOV - IRCCS, Padova, Italy; ^4^ IRCCS Ospedale Policlinico San Martino, Genova, Italy; ^5^ Istituto Europeo di Oncologia - IRCCS, Milano, Italy; ^6^ Plastic and Reconstructive Surgery Department, Università degli Studi di Bari Aldo Moro, Bari, Italy; ^7^ Clinica Dermatologica, Dipartimento di Scienze Mediche, Università di Torino, Torino, Italy; ^8^ Oncology Department, Ospedale Sant’Anna di Como, Como, Italy; ^9^ Fondazione I.R.C.C.S. Policlinico San Matteo, Pavia, Italy; ^10^ IRCCS Istituto Tumori “Giovanni Paolo II”, Bari, Italy; ^11^ Dermatology Department, Università di Firenze, Firenze, Italy; ^12^ Oncology Department, Azienda ospedaliera Santa Croce e Carle, Cuneo, Italy; ^13^ Mesothelioma Unit, Azienda Ospedaliera SS Antonio e Biagio e Cesare Arrigo, Alessandria, Italy; ^14^ Oncology Department, ospedale “Vito Fazzi” di Lecce, Lecce, Italy; ^15^ ULSS 2 Marca Trevigiana Ospedale Ca’ Foncello Treviso, Treviso, Italy; ^16^ Gruppo melanoma e tumori rari, Oncology Department, PO A Businco ARNAS G. Brotzu, Cagliari, Italy; ^17^ Dermatology Clinic, Department Medical Sciences and Public Health, University of Cagliari, Cagliari, Italy; ^18^ Oncology Department, Presidio Ospedaliero “Senatore Antonio Perrino”, Brindisi, Italy; ^19^ IRCCS Centro di Riferimento Oncologico Basilicata, Rionero in Vulture, Italy; ^20^ Dermatology Clinic, Università degli studi di Padova, Padova, Italy; ^21^ Centro di Riferimento Oncologico di Aviano (CRO) IRCCS, Aviano (PN), Italy; ^22^ Registro Tumori Provincia di Sassari, Azienda Ospedaliera Universitaria Sassari, Sassari, Italy; ^23^ Istituto di Ricerca Genetica e Biomedica, CNR, Sassari, Italy

**Keywords:** medical record systems, cutaneous melanoma, survival analysis, immunotherapy, ipilimumab

## Abstract

**Background:**

Cutaneous melanoma (CM) is one of the most aggressive types of skin cancer. Currently, innovative approaches such as target therapies and immunotherapies have been introduced in clinical practice. Data of clinical trials and real life studies that evaluate the outcomes of these therapeutic associations are necessary to establish their clinical utility. The aim of this study is to investigate the types of oncological treatments employed in the real-life clinical management of patients with advanced CM in several Italian centers, which are part of the Clinical National Melanoma Registry (CNMR).

**Methods:**

Melanoma-specific survival and overall survival were calculated. Multivariate Cox regression models were used to estimate the hazard ratios adjusting for confounders and other prognostic factors.

**Results:**

The median follow-up time was 36 months (range 1.2-185.1). 787 CM were included in the analysis with completed information about therapies. All types of immunotherapy showed a significant improved survival compared with all other therapies (p=0.001). 75% was the highest reduction of death reached by anti-PD-1 (HR=0.25), globally immunotherapy was significantly associated with improved survival, either for anti-CTLA4 monotherapy or combined with anti-PD-1 (HR=0.47 and 0.26, respectively) and BRAFI+MEKI (HR=0.62).

**Conclusions:**

The nivolumab/pembrolizumab in combination of ipilimumab and the addition of ant-MEK to the BRAFi can be considered the best therapies to improve survival in a real-world-population. The CNMR can complement clinical registries with the intent of improving cancer management and standardizing cancer treatment.

## Introduction

Cutaneous melanoma (CM) is one of the most aggressive types of skin cancer. The incidence of CM has increased in Europe over the last years, and cohort studies suggest that the increasing trend of incidence will continue for at least the next 2 decades (1–3) Mortality rates have also increased in the last decades, especially in men, despite a clear decrease of Breslow tumor thickness in the USA and Europe (1, 4). In the USA, the raw mortality rates per 100,000 inhabitants per year increased from 2.8 to 3.1, with an estimate of 10,130 deaths from melanoma in 2016 (they were 8,650 in 2009) (1). In Italy, 12,300 new cases and over 2,000 deaths were estimated in 2019 (5, 6).

Surgery is currently the golden standard for patients with early stage CM, who represent only part of the global cases. The treatment of patients with advanced stage CM is more complex, as for decades no chemotherapy regimens have been found effective in prolonging survival. Currently, innovative approaches such as target therapies and immunotherapies have been introduced in clinical practice for the treatment of metastatic CM. Target therapies are based on the use of drugs targeting specific genetic alterations in candidate genes, blocking specific pathways implicated in the oncogenesis of melanoma (7). BRAF mutations represent currently the main molecular targets for melanoma treatment, as they involve approximately 50% of the cases, and identify patients who may benefit from treatment with BRAF inhibitors, like vemurafenib or dabrafenib (8–10). Recently, the combination of BRAFi drugs with MEK inhibitors showed improved oncological outcomes in comparison to monotherapies (70% one-year and 50% two-years survival), with a better safety profile (11–13).

Immuno-therapy enhances the immune system’s T-cell response and indirectly affects cancer cells by stimulating the patient’s immune system (14). Ipilimumab, a monoclonal antibody that blocks the activity of the CTLA-4, has shown a long-term survival in about 20% of the patients treated (15–17). Programmed death 1 (PD1) is a membrane receptor of tumor cells (its main ligand is PD-L1) that represents a powerful brake to the immune system’s response and the target of specific inhibitors (nivolumab and pembrolizumab). Recently they have been introduced into clinical practice, as they were shown more effective than ipilimumab in terms of overall survival (OS) and toxicity (18, 19). Recent studies showed that the combination of anti-CTLA-4 and anti-PD-1 is more effective than monotherapy, but a higher incidence of high-grade adverse events was found (20). Combinations of targeted therapies and immunotherapies are currently investigated; the advantage of such combinations is that more than one anti-tumoral mechanism are employed against CM. Data of clinical trials and real life studies that evaluate the outcomes of these therapeutic associations are necessary to establish their clinical utility.

The aim of this study is to investigate the types of oncological treatments employed in the real-life clinical management of patients with advanced CM in several Italian centers which are part of the Clinical National Melanoma Registry (CNMR), and the oncological outcomes obtained.

## Materials and Methods

### Patients and Data Collection

CNMR is the first clinical registry established in Italy in 2010. It collects data from a wide network of melanoma centers throughout the country with the aim to carry out clinical and therapeutic evaluations investigating geographical and policy differences and instruments for planning specific health interventions in different populations and areas, in order to optimize the clinical management and survival of CM patients. CNMR collects data of patients with a histologically confirmed diagnosis of primary CM treated in 38 Italian institutions (hospitals, research institutes, ecc.) participating in the network, as previously described (21). The AJCC7 staging was used. For the purposes of the present study, data of consecutive patients enrolled from January 2011 to December 2018 were considered (CNMR established in 2010 but the first year was spent for administrative approvement and ethical committee in each centers).

**Figure 1:**
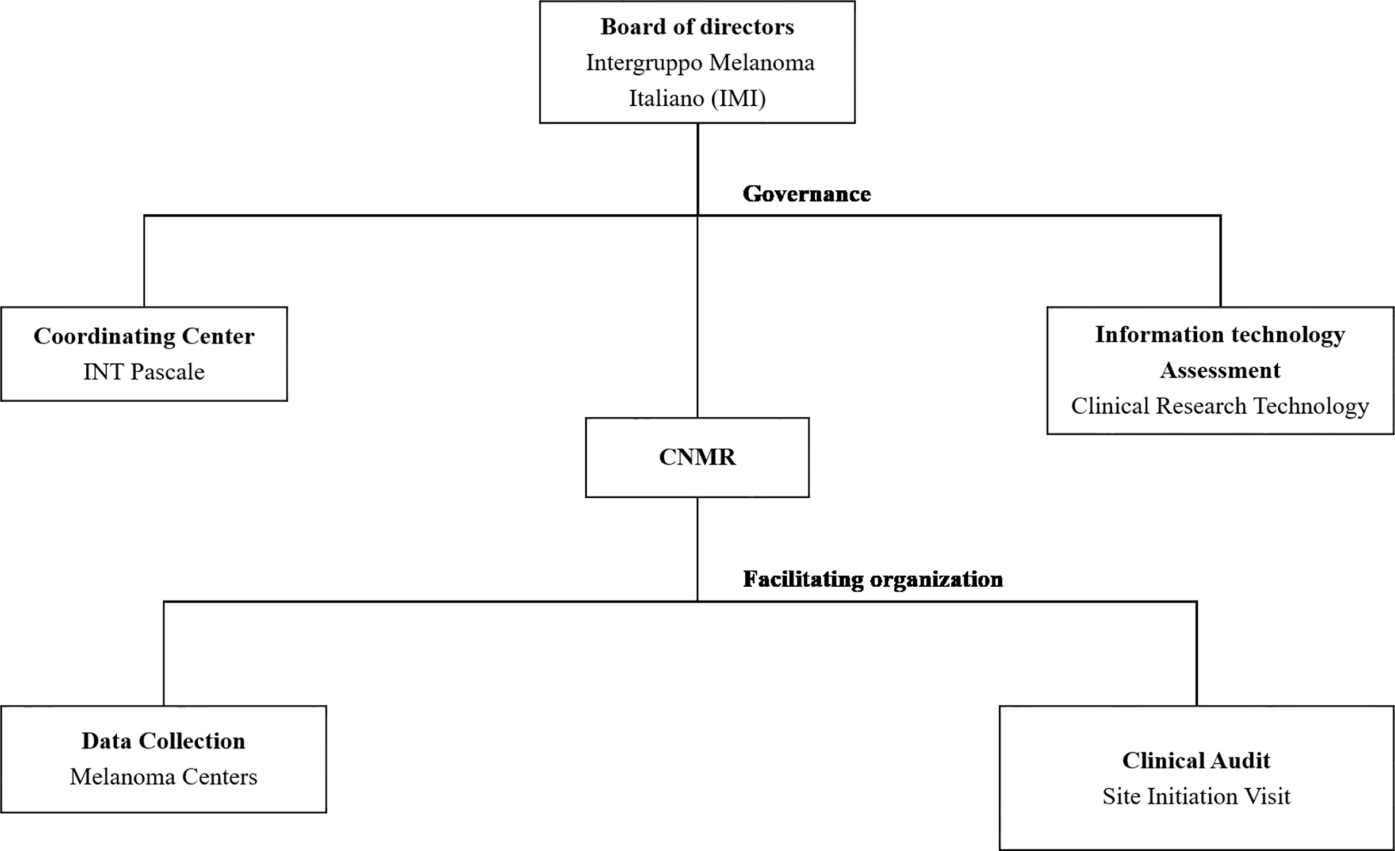
Integrated management of CNMR. Two main complementary approaches are considered—Governance and Facilitating organization.

A diagram of the CNMR’s organizational structure can be found in **Figure 1**.

Data were collected *via* an electronic Case Report Form (eCRF), which was developed by the Clinical Research Technology S.r.l. group (Salerno, Italy) on its clinical platform ‘eClinical’. ‘eClinical’ assigned an identification (ID) number to all the patients screened. The quality of the electronic data was verified through onsite clinical visits, undertaken periodically during the study. The eCRF was designed to collect information on sociodemographic, clinical, pathological and treatment variables. The first treatment was registered in all cases: local therapy (radiotherapy and electro-chemotherapy), systemic chemotherapy (platinum salts, dacarbazine, fotemustine), targeted therapy (BRAFi: vemurafenib/dabrafenib; BRAFI+MEKI: cobimetinib/trametinib), and immunotherapy (anti-CTLA4: ipilimumab, anti-PD-1: nivolumab/pembrolizumab; and anti-CTLA4 + anti-PD-1). Further information regarding the date of diagnosis, the duration of therapy, the date of the last follow-up, and the clinical status of the patients were also registered. Eligible patients for the survival analysis had histologically confirmed, unresectable stage III or stage IV metastatic melanoma (stage IIIB-IV) with an Eastern Cooperative Oncology Group (ECOG) performance status 0 or 3, and known BRAF mutation status.

### Statistical Analysis

Descriptive statistics for the categorical data were reported. Pearson’s Chi-squared was used to compare categorical variables. All patients were followed until 31 December 2018 or until the date of last visit, or death, whichever came first.

Melanoma-specific survival (MSS) was calculated from the date of initial adjuvant treatment to death for the disease and Overall survival (OS) until date of death from any cause. Patients who did not die were censored for OS on the last visit date available in the database. When the date of diagnosis was antecedent the beginning of the Melanoma Registry or the initial diagnosis was an early melanoma we considered the MSSurvival from the date of initial adjuvant treatment.

Kaplan-Meier curves and medians of OS and 95% CI are presented overall and by immunotherapy and target treatments. The Log-rank test compared curves by treatments (immunotherapy: anti-CTLA4, an-ti-PD-1 *vs.* no immunotherapy and no target therapy; BRAF: BRAFi, BRAFI+MEKI *vs.* no immunotherapy and no target therapy). Univariate and multivariable Cox regression models were used to estimate the hazard ratios adjusting for confounders and other prognostic factors.

All statistical tests were two-sided. P-values < 0.05 were considered significant. Statistical analyses were performed using statistical software SAS (version 9.02 for Windows), and Statistical Package for Social Science (SPSS) version 25 (SPSS inc., Chicago IL, USA).

## Results

Patients characteristics, sex, age, LDH, stage, BRAF execution and mutational status were reported in **Table 1**.

**Table 1:** Tumor characteristics for Advanced Stage (IIIB-IIIC unresectable, IV).

	ADVANCED STAGE
	IIIB-IIIC (*unresectable*), IV N=787
Gender^*^
*Male*	*476 (60)*
*Female*	*307 (39)*
*missing*	*4 (1)*
**Age**	
*≤60 yrs*	*355 (45)*
*>60 yrs*	*432 (55)*
**BMI**	
*<25*	315 (40)
*≥25*	386 (49)
*missing*	86 (11)
**LDH**	
*Normal*	479 (61)
*Abnormal*	43 (5)
*Unknown*	265 (34)
**Initial Stage**	
*In situ*	98 (12)
*Stage I-II*	297 (38)
*Stage III*	291 (37)
*Stage IV*	101 (13)
**BRAF executed**	
*No*	120 (15)
*Yes*	594 (76)
*Not applicable*	73 (9)
**BRAF mutational status**	
*Mutant*	322 (54)
*Wild Type*	269 (45.5)
unknown	3 (0.5)
***Mutant***	
***BRAF V600***	56 (17.4)
***BRAF V600E***	208 (64.6)
***BRAF V600K***	34 (10.6)
***Other***	24 (7.5)
***Year BRAF executed***	
<2013	498 (63)
≥2013	289 (37)

^*^4 patients did not report the gender.

Regarding to stage 12% had an initial diagnosis of “in situ”, 38% had an early diagnosis (IA-IIC), 37% stage III and 13% had a confirmed advanced melanoma stage (IV). 76% was the percentage of BRAF executed in our sample and the incidence of BRAF mutations was slightly greater than 50% and 65% reported a BRAF V600E mutation most cases were analyzed after the year 2013 when target therapies were diffusely employed in clinical practice; in addition, more cases among those analyzed harbored stage IV tumors rather than stage IIIB-IIIC melanomas.

The median follow-up time was 36 months (range 1.2-185.1). Observed patients and percentage according to type of treatment were reported in **Table 2**; total death events (for all causes and deaths for the diseases) were reported and median Melanoma-specific survival (MSS) were calculated. As first line of treatment (choice), 41% of patients (n=319) received immunotherapy, 36% received BRAF-targeted therapies (n=285), 35% received chemotherapy (n=275) and 35% received local therapy (electrochemotherapy) (n=275). In details, among immunotherapy: 62% received ipilimumab (anti-CTLA4), 25% nivolumab/pebrolizumab (anti PD1), 13% the two combined. Among BRAF therapy: 69% received BRAFi as monotherapy (vemurafenib/dabrafenib), about 31% received BRAFi+MEK combination treatment (vemurafenib/dabrafenib + cobimetinib/trametinib).

**Table 2:** Distribution of therapies and combined therapies in the cohort of advanced melanoma patients.

Indicator	Advanced Melanoma: IIIB-IIIC (unresectable), IV	
	*Observed patients (n)*	*(%)*
Patients eligible for analysis	787	(100)
Patients with at least one local treatment	275	(35)
No local treatment	512	
Patients with at least one chemotherapy	275	(35)
No chemotherapy	512	
Patients with at least one immunotherapy	319	(41)
Immunotherapy: ANTI-PD-1 (Nivolumab/Pebrolizumab)	*80 (25.1)*	
Immunotherapy: ANTI-CTLA4 (Ipilimumab)	*198 (62.1)*	
Immunotherapy: ANTI-PD-1 + ANTI-CTLA4	*41 (12.8)*	
No immunotherapy	468	
Patients with at least one target therapy (BRAFi, BRAFI+MEKI)	285	(36)
BRAFi: vemurafenib/dabrafenib	*198 (69.5)*	
BRAFI+MEKI: cobimetinib/trametinib	*87 (30.5)*	
No target therapy	502	
Numebr of Line-therapies		
LineI°	*233 (29.6)*	
Line I°+II°	*238 (30.2)*	
LineI°+II°+III°	*316 (40.2)*	

In the entire cohort the median overall melanoma-specific survival was 47 months (95% CI: 41-53), the lowest median survival was detected by patients treated by chemotherapy (33 months, 95% CI 27-38) as first option. Among immunotherapy the MSS globally was 50 months (95% CI 43-57), it varied from 47 months (95% CI 37-56) for ipilimumab (anti-CTLA4) to 70 months (95% CI 39-101) for nivolumab/pebrolizumab (anti-PD-1). Targeted therapy globally produced MSS of 44 months (95% CI 38-50), it varied from 40 months (95% CI 34-45) for BRAFi to 55 months (95% CI 49-61) for BRAFi+MEK (see **Table 3**).

**Table 3:** Results of the performance indicators on the quality of metastatic melanoma care – Univariate Analysis.

Long-term outcomes	Advanced Melanoma:IIIB-IIIC (unresectable), IV	
	Events^1^ (n) DOD/DEAD	Median MSS (95% CI)
***Melanoma-specific Survival (MSS) overall***	314/353	*47 (41-53)*
***Melanoma-specific Survival (MSS) of pts. with local treatment***	132/147	*42 (35-48)*
***Melanoma-specific Survival (MSS) of pts. with chemotherapy***	151/163	*33 (27-38)*
***Melanoma-specific Survival (MSS) of pts. with immunotherapy***	126/137	*50 (43-57)*
***MSS* Immunotherapy: ANTI-PD-1 (Nivolumab/Pebrolizumab)**	17/18	*70 (39-101)*
***MSS* Immunotherapy: ANTI-CTLA4 (Ipilimumab)**	94/104	*47 (37-56)*
***MSS* Immunotherapy: ANTI-PD-1+ANTI-CTLA4**	15/15	*58 (26-90)*
***Melanoma-specific Survival (MSS) of pts. with* target therapy**	129/147	*44 (38-50)*
***MSS* BRAFi: vemurafenib/dabrafenib**	91/107	*40 (34-45)*
***MSS* BRAFI+MEKI: cobimetinib/trametinib**	38/40	*55 (49-61)*

^1^Event: number of deaths of the disease (DOD)/number of deaths for all causes (DEAD).

**Figure 2:**
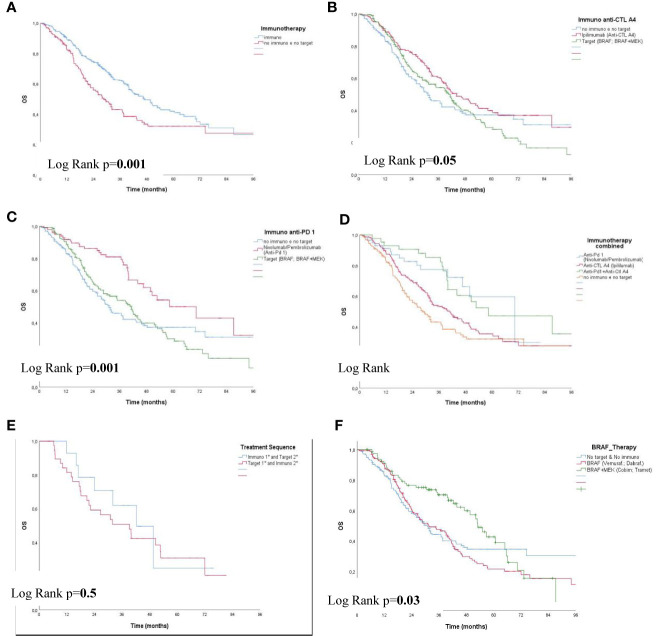
Overall Survival (OS) in patients with IIIB-IIIC (UNRESECTABLE), IV by Therapy **(A–F)**. **(A)** Overall Survival (OS) Immunotherapy, **(B)** OS Immunotherapy: ANTI-CTL A4, **(C)** OS Immuno: ANTI-PD 1, **(D)** OS Immuno: ANTI-PD 1; ANTI-CTLA4; ANTI PD 1+ANTI-CTL A4, **(E)** OS Treatment Sequence:Immuno 1st, 2nd; Target 1st, 2nd; Target 1st & Immuno 2nd **(F)** OS BRAF *vs.* BRAFI+MEKI.

Immunotherapy showed an improved survival compared with all other therapies (Chemotherapy, Local therapy and no targeted therapy) (p=0.001) (**Figure 2A**); for Ipilimumab and combined target therapy compared with all other therapies a slight significance were observed (p=0.05) (see **Figure 2B**). The highest survival (70 months; 95% CI 45-96) was reached by patients treated with Nivolumab/Pembrolizumab compared with combined target therapy and all other therapies (p=0.001) (see **Figure 2C**); Immunotherapy across strata showed an improved survival for anti-PD-1 and combined anti-PD-1 + anti-CTLA4 compared with Ipilimumab and all other therapies (p<0.0001) (see **Figure 2D**). The treatment-sequence did not show any significant difference (Immuno in 1st and Target in 2nd *vs.* Target in 1st and Immuno in 2nd line) (p=0.5) (see **Figure 2E**). A significant difference was observed between BRAF *vs.* BRAF with the addition of Cobimetinid/Trametinib (anti-MEK) (p=0.03) (see **Figure 2F**).

Multivariate Cox model hazard ratios were reported in **Table 4**: a significant increased risk of death was observed for abnormal LDH compared to normal (HR=1.94 95% CI 1.23-3.06); among the Target therapy a significant protective effect was observed for target therapy with the addition of Cobimetinid/Trametinib (BRAFI+MEKI) (HR=0.63 95% CI 0.42-0.94). All immunotherapy categories were significantly associated with a reduction of death: anti-PD-1 HR=0.25 (95% CI 0.15-0.43), anti-CTLA4 HR=0.47 (95% CI 0.33-0.67) and combined anti-PD-1+ anti-CTLA4 HR=0.26 (95% CI 0.15-0.47), respectively. The treatment-sequence was not associated to the risk of death (p=0.3).

**Table 4:** Multivariate Cox regression models for death.

Parameter/Category	Adjusted Multivariate Analysis^‡^		
	HR	95% CI	p
***Gender***			
Female	1†		
Male	1.121	0.898-1.398	0.314
**Age**			
≤60	1†		
>60	1.192	0.961-1.478	0.109
**Area of enrollment in Italy^3^**			
Center/South	1†		
North	0.981	0.778-1.238	0.873
***Year BRAF executed^4^***			
<2013	1†		
≥2013	1.06	0.837-1.355	0.609
**LDH**			
*Normal*	1.0^†^		
*Abnormal*	1.95	1.24-3.01	0.004
*Unknown*	0.97	0.95-1.53	0.09
**Target therapy**			
*No Target and No Immuno therapy*	1.0^†^		
*BRAF*	1.14	0.85-1.53	0.4
*BRAFI+MEKI*	0.623	0.42-0.94	0.02
**Immunotherapy**			
*No Immuno and No Target therapy*	1.0^†^		
*ANTI-PD-1 (Nivolumab/Pebrolizumab)*	0.25	0.147-0.43	<0.0001
*ANTI-CTLA4 (Ipilimumab)*	0.47	0.33-0.67	<0.0001
*ANTI-PD-1+ ANTI-CTLA4*	0.26	0.15-0.47	<0.0001
**Treatment Sequence**			
*Immuno 1^st^ and Target 2^nd^*	1.0^†^		
*Target 1^st^ and Immuno 2^nd^*	1.64	0.65-4.12	0.3

^†^Reference category; ^‡^Multivariate Cox model adjusted for gender (male, female); age (≤60, >60); geographical area (North, Central-South);Year BRAF executed (≤2013, >2013); N. @ of therapies (1, 2, ≥3); Other therapies: Chemotherapy; Local and systemic therapy whenever.

## Discussion

In this study, we examined data of advanced melanoma in the Italian Clinical National Melanoma Registry (CNMR). CNMR does not have the typical aim of cancer registries to estimate incidence data, but as a clinical registry may collect data from the real world experience which is different from that coming from clinical studies which included selected patients (22, 23). Indeed, much of the existing research on advanced melanoma patients has been conducted in clinical trials settings among patients who meet stringent inclusion and exclusion criteria.

The analysis of the 787 patients from the advanced cohort showed some interesting results. As first, looking at the advanced patients’ characteristics, a good percentage of them come from the initial stages more than from the high risk conditions. Indeed, 50% of advanced melanoma had an initial diagnosis of early stage that then developed into advanced one.

Unfortunately, the BRAF mutational status was not evaluated in all patients; indeed, the BRAF status has been documented in as much as 76% of these patients. An important consideration is that the CNMR collected data from December 2011 and the most important drug in the field of melanoma, like BRAF inhibitors, anti-CTLA4, anti-PD-1 were approved in the following years. Specifically ipilimumab was the first treatment to be approved, on February 2013, by AIFA (The Italian Medicines Agency). Vemurafenib and dabrafenib received approval on May 2013 and on October 2014 respectively as monotherapy, and on September 2016 and on January 2017 in combination with cobimetinib and trametinib respectively. Pembrolizumab was approved on May 2016 while nivolumab on 24 March 2016 (24). Moreover, the possibility to ask for the BRAF mutational status was probably related only to the centers which were participating to clinical studies or expanded access programs with such drugs.

Study strengths include a large sample size, many treatment options reported (immunotherapy such as anti-PD-1 or combination of anti-PD-1 and anti-CTLA-4, or targeted therapies) and this is the first study investigating oncological treatments in a real-life clinical settings in advanced melanoma in several Italian centers. Study limitations include a lack of information like the metastatic site and the collection of therapy data was not completely reported, therefore the evaluation of the combined treatment (chemotherapy and immunotherapy/chemotherapy and targeted therapy) was not possible.

Concerning the OS, with some limitations due to the time of data collection (before the approval and the use of anti-PD-1 and BRAF/MEK inhibitors, and the small number of patients considered), there are still some interesting findings. It is evident that the new therapies available had an important impact on the survival of these patients. Indeed, patients who practiced immunotherapy or target therapy performed better in terms of median survival than those who practiced local therapy and/or chemotherapy, considered for a long time the only standard of treatment for metastatic melanoma. The addition of the MEK inhibitor to the BRAF inhibitor significantly improved patient OS.

It seems that the greater advantage in terms of OS is in those patients who have performed immunotherapy lines, even compared to those who have performed target therapies. This finding could be explained by the fact that many patients received BRAF inhibitor therapy as single agent (69,5%), and only a minority had benefit from the addition of the MEK inhibitor. Indeed, we learned that disease progression during therapy with the BRAF inhibitor alone was often rapid and unresponsive to subsequent treatments (25); with the addition of MEK inhibitors, the fast progression from target therapy was reduced (26).

The data on the combination nivolumab + ipilimumab also appears intriguing, especially in terms of long survival; however, the low number of patients does not allow us to give definitive conclusions.

The correlation between survival and the LDH value is also consistent with the literature data. Analyzing the LDH values, there is an increased risk of death for patients with high LDH, compared to those with normal LDH, especially in the group of patients who received immunotherapy (HR = 2.45, p = 0.01)

We found that immunotherapy allows better results in terms of overall survival in patients with advanced melanoma, however in our analysis there is no statistically significant benefit of the treatment-sequence variable (Immuno in 1st and Target in 2nd *vs.* Target in 1st and Immuno in 2nd line). In consideration of the retrospective analysis, the small number of patients who started with anti-PD-1, and the lack of patients who received the dual MAPK blockade, definitive conclusions cannot be made.

At the moment several combination studies of target and immunotherapies as well as protocols to establish the best sequential therapy are ongoing (27). Our study has several limitations. In fact, most patients received chemotherapy as a first systemic treatment for advanced disease, because more effective drugs such as BRAF/MEK inhibitors, anti-CTLA4 and anti-PD-1 inhibitors were approved subsequently in different years. In addition, many centers did not test all patients for BRAF, especially at the beginning.

## Conclusions

Finally, this study shows that immunotherapy improves survival in advanced melanoma in a real-world population. The CNMR represents a set of data useful not only to plan the appropriate prevention measures but to better understand the effectiveness of anti-cancer treatments in a large unselected population from a real world experience. Furthermore, qualified data is essential and it is important that this information is constantly updated in order to maintain high levels of evidence.

The nivolumab/pembrolizumab and the combination of ipilimumab can be considered the best therapy to improve survival in a real-world-population. The CNMR can complement clinical registries with the intent of improving cancer management and standardizing cancer treatment.

## Data Availability Statement

The datasets presented in this study can be found in online repositories. The names of the repository/repositories and accession number(s) can be found below: http://imi.cr-technology.com/cnmr.

## Ethics Statement

CNMR was approved by ethical committee of Istituto Nazionale dei Tumori Fondazione G. Pascale, protocol n.10/10, prot. CEI 537/10. The patients/participants provided their written informed consent to participate in this study.

## CNMR Group

**Maddalena Cespa**, Fondazione I.R.C.C.S. Policlinico San Matteo Clinica Dermatologica, Pavia: **Rosachiara Forcignanò**, Azienda Ospedaliera Vito Fazzi, U.O. Di Oncologia, Lecce; **Gianmichele Moise**, Azienda Per I Servizi Sanitari N°2 Isontina Ospedale Di Gorizia Dipartimento Di Medicina , S.O.S. Di Dermatologia –Gorizia; **Maria Concetta Fargnoli**, Presidio Ospedaliero San Salvatore, U.O.S. Di Dermatologia Generale Ed Oncologica, L’Aquila; **Caterina Ferreli**, Università Degli Studi Di Cagliari - Azienda Ospedaliero Universitaria, Clinica Dermatologica, Cagliari; **Maria Grimaldi**, Istituto Nazionale dei Tumori Fondazione G. Pascale Napoli; **Guido Zannetti**, Azienda Ospedaliero-Universitaria Di Bologna Policlinico S. Orsola -Malpighi, Chirurgia Plastica, Bologna; **Saverio Cinieri**, Presidio Ospedaliero Antonio Perrino, U.O.C. Di Oncologia E Breast Unit, Brindisi; **Giusto Trevisan**, Ospedale Maggiore, Azienda Ospedaliera Universitaria Di Trieste, Clinica Dermatologica ,4° Piano (Palazzina Infettivi), Trieste; **Ignazio Stanganelli**, Ospedale S.Maria Delle Croci - Usl Di Ravenna, Centro Di Dermatologia Oncologica CPO/IRST, Ravenna; **Giovanna Moretti**, Azienda Ospedaliera Ospedali Riuniti Papardo-Piemonte S.C. Dermatologia Messina; **Francesca Bruder**, Ospedale Oncologico, Dipartimento Melanoma E Tumori Rari 5° Piano, Cagliari; **Luca Bianchi**, Azienda Ospedaliera Universitaria Policlinico Tor Vergata U.O.C. Dermatologia, Roma; **Maria Teresa Fierro**, A.O.U. Città Della Salute E Della Scienza - P.O. San Lazzaro, S.C. Dermatolgia Torino; **Luigi Mascheroni**, Humanitas - Casa Di Cura San Pio X S.R.L., Chirurgia GeneraleMilano; **Salvatore Asero**, Azienda Ospedaliera Di Rilievo Nazionale E Di Alta Specializzazione Garibaldi-Nesima, U.O. Di Chirurgia Oncologica - Dip. Oncologia, Catania; **Caterina Catricalà**, Istituto Dermatologico San Gallicano IRCCS – IFO, UOC di Dermatologia Oncologica - Dipartimento Clinico-Sperimentale Di Dermatologia Oncologica Roma; **Stefania Staibano**, Azienda Ospedaliera Universitaria Federico II di Napoli, Scienze Biomorfologiche e Funzionali-Sezione Di Anatomia Patologica, Napoli; **Gaetana Rinaldi**, Azienda Ospedaliera Universitaria Policlinico `Paolo Giaccone`,Dipartimento Di Oncologia - U.O.C. Oncologia Medica, Palermo; **Riccardo Pellicano**, IRCCS Casa Sollievo Della Sofferenza, U.O.C. Dermatologia, San Giovanni Rotondo; **Laura Milesi**, Azienda Ospedaliera Papa Giovanni XXIII, USC Oncologia Medica, Bergamo; **Marilena Visini**, A.O. Di Lecco Presidio Ospedaliero Alessandro Manzoni, Oncologia Medica, Lecco; **Franco Di Filippo**, Istituto Nazionale Tumori Regina Elena IRCCS – IFO, Chirurgia Generale A, Roma; **Leonardo Zichichi**, Azienda Sanitaria Provinciale - Presidio Ospedaliero Di Trapani, U.O. C. Dermatologia, Casa Santa – Erice; **Maria Antonietta Pizzichetta**, Centro Di Riferimento Oncologico, Istituto Nazionale Tumori, Divisione Di Oncologia Medica C, Aviano; **Carmelo Iacono**, Azienda Ospedaliera Sanitaria 7 Ragusa - Ospedale Maria Paternò Arezzo, Dipartimento Di Oncologia, Ragusa; **Massimo Guidoboni**, I.R.S.T. Istituto Scientifico Romagnolo Per Lo Studio E La Cura Dei Tumori U.O. Immunoterapia E Terapia Cellulare Somatica, Meldola; **Giovanni Sanna**, Azienda Ospedaliero-Universitaria Di Sassari, Servizio Di Medicina Nucleare U.O. Di Oncologia Medica, Sassari; **Michele Maio**, Azienda Ospedaliera Universitaria Senese Ospedale Le Scotte U.O.C. Immunoterapia Oncologica, Siena; Michele Del Vecchio, Fondazione I.R.C.C.S. Istituto Nazionale Dei Tumori, S.C. Medicina Oncologica 1, Milano; **Lucia Lospalluti**, Azienda Sanitaria Locale BA - Ospedale Di Venere, U.O. Dermatologia, Carbonara Di Bari; **Rosanna Barbati**, Asl Roma C - Ospedale S.Eugenio , U.O. Dermatologia, Roma; **Leonardi Vita**, ARNAS Civico Palermo; **Annamaria Pollio**, Ospedale “A. Cardarelli” – Campobasso, U.O.C. di Anatomia Patologica; **Carlo Riberti**, Istituto di Chirurgia Plastica presso l’Arcispedale Sant’Anna, Ferrara.

## Author Contributions 

Conceptualization, AC, GP, AT, and PA. Methodology, AC, VV, MB, SG, GP, and PA. Software, AC, and SG. Validation, AC, MC, AV, PF, PQ, FS, VV, CC, GT, GM, EC, MB, SG, AT, GP, and PA. Formal analysis, AC, VV, and SG. Investigation, MC, EG, AV, PF, PQ, FS, VV, CC, GT, EP, GG, EN, PQ, SR, MiG, DaM, SB, MoG, VG, MO, FG, GC, AG, DeM, LA, NC, TF, GM, FT, EC, MB, SG, CR, AT, and PA. Resources AC, VV, MB, SG, GP, and PA. Data curation, AC, VV, GP, and SG. Writing—original draft preparation, AC, MC, AV, PF, PQ, FS, VV, CC, GT, GM, EC, MB, SG, AT, GP, and PA. Writing—review and editing, AC, VV, GT, GM, EC, MB, SG, AT, GP, and PA. Visualization, AC, VV, SG, GP, and PA. Supervision, AC, VV, MB, SG, GP, and PA. Project administration, AC, VV, MB, SG, GP, and PA. Funding acquisition, CR, AT, and PA. All authors contributed to the article and approved the submitted version.

## Funding

This research was funded by grants received from Bristol Myers Squibb (New York, NY, USA), GlaxoSmithKline (Brentford, UK) and Pierre Fabre Pharma. The funders were not involved in the study design, collection, analysis, interpretation of data, the writing of this article or the decision to submit it for publication.

## Conflict of Interest

PA has/had a consultant/advisory role for Bristol Myers Squibb, Roche-Genentech, Merck Sharp & Dohme, Novartis, Array, Merck Serono, Pierre-Fabre, Incyte, Medimmune, AstraZeneca, Syndax, Sun Pharma, Sanofi, Idera, Ultimovacs, Sandoz, Immunocore, 4SC, Alkermes, Italfarmaco, Nektar, Boehringer-Ingelheim, Eisai, Regeneron, Daiichi Sankyo, Oncosec, Pfizer. He also received research funding from Bristol Myers Squibb, Roche-Genentech, Array and travel support from MSD.

The remaining authors declare that the research was conducted in the absence of any commercial or financial relationships that could be construed as a potential conflict of interest.
